# **The impact of vividness of visual imagery on the construction of multi-dimensional situation models by second language learners**

**DOI:** 10.1007/s10339-025-01304-6

**Published:** 2025-09-26

**Authors:** Ran Tang, Qichao Song, Norio Matsumi

**Affiliations:** 1 School of Foreign Languages, Tianjin Chengjian University, Tianjin, China; 2 School of Japanese and International Studies, Beijing Centre for Japanese Studies, Beijing Foreign Studies University, Beijing, China; 3 Graduate School of Humanities and Social Sciences, Hiroshima University, Hiroshima, Japan

**Keywords:** Second-language reading comprehension, Event-indexing model, Multidimensional situation model construction, Visual imagery vividness

## Abstract

This study investigates how Chinese native speakers learning Japanese as a second language construct situation models across five dimensions, i.e., protagonist, time, space, causality, and intentionality, while reading Japanese narratives, and how visual imagery vividness affects this process. Employing a generalized linear mixed-effects model, we conducted an analysis of verb-clustering data. The results showed that (1) the protagonist, time, and space dimensions played significant roles in constructing situation models in Chinese learners of Japanese, while the causality and intentionality dimensions did not have significant impacts; (2) the construction of situation models in L2 learners’ reading was influenced by visual imagery vividness. Learners with higher visual imagery vividness were better able to construct accurate situation models when the protagonist and space dimensions were discontinuous. The findings provide new insights into understanding the cognitive processing mechanisms of second-language learners and suggest that visual imagery vividness plays a crucial role in the construction of situation models. This research offers empirical support for optimizing teaching strategies of second-language reading.

## Introduction

Understanding narrative texts in a second language (L2) plays a critical role in overall language development. Investigating the cognitive mechanisms underlying L2 reading processes can inform the optimization of reading instruction and contribute to the development of effective methodologies for foreign language learning (e.g., Pretorius et al. 2022; Sun et al. 2024; Yang et al. 2023). Compared to first language (L1) reading, L2 reading imposes greater cognitive demands, as it requires learners to coordinate linguistic decoding with higher-level discourse processing and cross-linguistic transfer (Van Gelderen et al. 2004). Although interest in L2 reading comprehension has grown in recent years, most existing studies have focused on learners of English as an L2, such as L1 Spanish–L2 English, L1 Chinese–L2 English or L1 Japanese–L2 English combinations (e.g., Ogiso et al. 2023; Perry 2013; Sun et al. 2024; Tsai et al. 2010; Ushiro et al. 2020; Ushiro et al. 2022). In contrast, studies examining narrative processing in L2 contexts involving two Asian languages—such as L1 Chinese and L2 Japanese—remain comparatively scarce (e.g., Tang 2024; Zhang 2022). These language pairings pose unique challenges due to differences in cognitive load, script systems, and discourse conventions (Cai et al. 2025; Song et al. 2023). Investigating how learners process narrative information in this context provides both theoretical insight and practical relevance.

Among the many challenges encountered in L2 reading, one of the most significant is achieving deep comprehension, which depends on the ability to construct a coherent mental representation of the text (e.g., Ushiro et al. 2020; Zwaan and Brown 1996). According to situation model theory, readers form multidimensional mental representations of narrative events by integrating information along five core dimensions: protagonist, time, space, causality, and intentionality (Zwaan et al. 1995b; Zwaan and Radvansky 1998). These representations go beyond surface-level information and reflect a reader’s ability to understand how events are interconnected. While prior research has shown that L1 readers actively monitor and update situational dimensions during reading, much less is known about whether L2 learners construct situation models in a similar way. In addition to this gap in understanding L2 readers’ construction processes, it also remains unclear whether individual cognitive factors—such as the vividness of visual imagery—affect their sensitivity to these situational dimensions.

To address these gaps, the present study examines whether Chinese learners of Japanese construct coherent situation models along the five core dimensions, and whether individual differences in visual imagery vividness modulate this process. In doing so, the study contributes to a deeper understanding of the cognitive mechanisms underlying situation model construction in L2 reading.

## Literature review

### Event indexing model and its measurement

Narrative comprehension involves constructing a coherent situation model in which readers represent how each new event relates to preceding events along five core dimensions: protagonist, time, space, causality, and intentionality (e.g., Zwaan et al. 1995b; Zwaan 2016). The protagonist dimension refers to the central characters involved in an event; the time and space dimensions establish the temporal and spatial contexts; the causality dimension captures cause–effect relationships; and the intentionality dimension reflects the goals and plans of the protagonists. This theoretical framework, known as the Event Indexing Model (EIM), is built on four core assumptions: (1) comprehension requires constructing a coherent situation model in which each new sentence is integrated with prior story context; (2) readers continuously monitor these dimensions during reading; (3) discontinuities along any dimension impose additional cognitive demands and trigger updates to the mental model; and (4) narrative events are encoded in memory in accordance with their degree of continuity across these dimensions (Zwaan and Radvansky 1998).

A seminal contribution by Zwaan et al. (1995b) provided some of the earliest and most direct evidence for the EIM. Using the verb-clustering task, they found that participants tended to group verbs from narratives according to shared situational dimensions—specifically, time, space, protagonist, causality, and intentionality—demonstrating that continuity across these five dimensions guides the organization of events in readers’ mental representations. In this paradigm, participants are presented with a list of verbs from a narrative after reading and are asked to group them based on perceived similarity. These subjective groupings reflect readers’ mental representations of event structure and typically correspond to continuity along specific situational dimensions. For example, the verbs in “The teacher entered the classroom” and “The teacher began teaching” are likely to be grouped together, as they share the same protagonist and spatial setting, thus forming a cohesive unit in the reader’s mental model.

The verb-clustering task offers two key advantages: (1) it enables the simultaneous assessment of multiple situational dimensions (Zwaan et al. 1995b); and (2) it allows for the use of ecologically valid texts, such as actual novels and essays, rather than artificially constructed passages (Wada 2019). Accordingly, this paradigm not only provided early and direct evidence for the EIM but also laid the methodological foundation for much of the subsequent research cited below.

Building on this foundation, a substantial body of research has offered further support for the core assumptions of the model. Studies of online processing have shown that readers typically slow down when encountering breaks in situational continuity, such as changes in protagonist, time, or space, suggesting that additional cognitive effort is required to update the mental model (e.g., Radvansky and Copeland 2010; Rinck and Weber 2003; Rich and Taylor 2000; Speer and Zacks 2005; Therriault et al. 2006; Zwaan et al. 1995a, 1998). Other lines of evidence indicate that events are represented in episodic memory as interconnected nodes, with the strength of their associations determined by the degree to which they share situational dimensions (e.g., Bohn-Gettler et al. 2011; Iseki and Kawasaki 2006; Wada 2019; Zwaan and Brown 1996). The verb-clustering task introduced by Zwaan et al. (1995b) has since been widely adopted in this literature, offering an effective means of examining how multiple situational dimensions are simultaneously represented (e.g., Bohn-Gettler et al. 2011; Iseki and Kawasaki 2006; Takaki 2010; Wada 2019). Following this tradition, the present study employs the verb-clustering task to investigate how L2 readers construct and update situation models during narrative comprehension.

### Construction of situation model in L2 reading

Research on L1 reading has shown that native speakers routinely monitor and update multiple situational dimensions during narrative comprehension (e.g., Iseki and Kawasaki 2006; Radvansky and Copeland 2010; Zwaan et al. 1995a, 1998), providing a useful benchmark for examining L2 readers. In contrast, studies on L2 reading suggest that while learners are capable of constructing situation models, they tend to monitor a more limited set of dimensions (e.g., Takaki 2010; Ushiro et al. 2020; Zwaan and Brown 1996). Zwaan and Brown (1996) were the first to apply the verb-clustering task in an L2 context. Their results showed that native English speakers reading French as an L2 primarily tracked the dimensions of protagonist, time, and causality. In contrast, during L1 reading, they significantly monitored time, causation, motivation, and protagonist. This pattern suggests that L2 readers monitor situational dimensions more selectively, likely due to limited proficiency and increased processing demands.

Subsequent research has both extended and refined these findings. Takaki (2010) found that Japanese high school students monitored the protagonist and spatial dimensions when reading English texts, while Ushiro et al. (2020) reported that Japanese learners of English relied more heavily on conscious, strategic processes to establish protagonist links and encountered greater difficulty with temporal and spatial shifts. Such variation across studies may reflect differences in learners’ proficiency and reading experience. Taken together, these studies confirm that L2 readers are capable of constructing multidimensional situation models, but their engagement with situational dimensions is often more restricted than that of native speakers. Moreover, the results are consistent with Zwaan and Brown’s (1996) observations, suggesting that sensitivity to situational dimensions may also depend on the specific L1–L2 pairing, thereby underscoring the need for further research in less-studied language combinations.

### Factors influencing the construction of situation models

Construction of situation models is influenced by a complex interplay of both verbal and nonverbal factors. Regarding verbal influences, research in L1 contexts has shown that text genre (e.g., narrative versus expository) significantly affects the activation of situational dimensions (Iseki and Kawasaki 2006). In L2 reading, verbal factors play an even more critical role: higher L2 proficiency is associated with the construction of richer and more coherent situation models (Takaki 2010), while text genre (Maeda 2017) and reading instructions (Zhang 2022) further facilitate the development of situation models.

Beyond these linguistic factors, a growing body of research highlights the pivotal role of perceptual simulation and mental imagery in narrative comprehension. According to the immersed experiencer framework—an embodied theory of language comprehension—readers mentally simulate described events by drawing on prior sensory and motor experiences (Zwaan 2004). Empirical studies have demonstrated that readers spontaneously generate dynamic mental images—such as tracking object shapes, orientations, or narrative scenes as narratives unfold. These mental images facilitate the incremental construction and updating of coherent situation models both in L1 (Stanfield and Zwaan 2001; Zwaan and Pecher 2012; Zwaan et al. 2002) and in L2 (Shiang et al. 2024; Tang 2023). Conversely, suppressing mental imagery has been shown to impair the formation of situation models (Fincher-Kiefer 2001).

Furthermore, external visual supports, such as illustrations or imagery instructions, can facilitate the construction of situation models. Illustrations, for example, have been shown to improve the monitoring of causal relations in L1 contexts (Wada 2019). In addition, imagery instructions enhance spatial and protagonist coherence, with research indicating their effectiveness in L2 contexts as well (Ushiro et al. 2022; Ogiso et al. 2023), although their effects on temporal processing appear more limited. Similarly, individual differences in visual imagery vividness have been shown to influence reading comprehension and are commonly assessed using the Vividness of Visual Imagery Questionnaire (VVIQ; Marks 1973). The VVIQ is a widely used self-report measure designed to assess the subjective vividness of mental imagery, including the clarity, richness, and perceptual detail of imagined scenes (Zhang et al. 2024 preprint). Higher VVIQ scores have been associated with richer discourse processing and better narrative recall in L1 contexts (Denis 1982, 1987; Hatakeyama 2018), suggesting that individuals with more vivid mental imagery may benefit from enhanced simulation during reading.

Although research in L2 contexts remains limited, Tang (2024) provides compelling empirical support, demonstrating that Chinese learners of Japanese with higher visual imagery vividness achieved superior comprehension and retention when instructed to visualize the text. This finding not only underscores the theoretical relevance of imagery vividness in L2 reading, but also validates the use of the VVIQ as a meaningful individual difference measure in this domain. However, most existing studies have focused primarily on memory and comprehension outcomes, leaving open the question of how visual imagery vividness contributes to the construction of multidimensional situation models during reading. This gap underscores the need for further investigation into the cognitive mechanisms linking imagery vividness to situational dimension monitoring in L2 contexts.

### Summary of existing literature and remaining issues

In summary, a substantial body of research has demonstrated that L1 readers construct situation models grounded in situational continuity, with verb-clustering tasks effectively revealing how narrative events are mentally represented and organized. Evidence also indicates that L2 learners tend to monitor fewer situational dimensions compared to L1 readers. While a few studies suggest that visual imagery vividness positively influences L2 reading comprehension, its specific role in the construction of multidimensional situation models remains underexplored.

Nonetheless, two critical gaps persist in the existing literature. First, few studies have investigated how L2 readers construct multidimensional situation models, particularly in the context of Chinese (L1)–Japanese (L2), a language pairing that presents both theoretical significance for discourse processing and practical implications for L2 reading pedagogy. Second, the influence of individual differences in visual imagery vividness on the construction of multidimensional situation models has not been systematically examined.

## Objectives and hypotheses

This study aims to address the aforementioned gaps by examining the dimensional structure of situation models constructed by Chinese learners of Japanese and investigating how individual differences in visual imagery vividness influence this process. To achieve this, we adopt a verb-clustering paradigm (e.g., Bohn-Gettler et al. 2011; Iseki and Kawasaki 2006; Takaki 2010; Wada 2019; Zwaan et al. 1995b), which captures how readers mentally organize narrative events along situational dimensions during L2 reading. In addition, we employ the Chinese version of the VVIQ (Zhang et al. 2024, preprint) to assess whether learners with more vivid mental imagery construct multidimensional situation models more effectively. Specifically, this study addresses the following research questions:

### Research question 1 (RQ1)

How do Chinese learners of Japanese construct multidimensional situation models during narrative reading in L2 Japanese?

### Research question 2 (RQ2)

To what extent does visual imagery vividness modulate the construction of situation models among Chinese learners of Japanese?

Based on the theoretical frameworks of the EIM and grounded cognition, we formulate the following two core hypotheses, along with their specific predictions for the current study:

***Event Indexing Hypothesis***

According to the EIM, events that share situational dimensions in a narrative are related in long-term memory along those dimensions (Zwaan et al. 1995b). This implies that readers track continuity across dimensions such as protagonist, time, space, causality, and intentionality during comprehension. Prior research suggests that L2 readers often monitor fewer dimensions than native speakers (e.g., Takaki 2010; Zwaan and Brown 1996), likely due to limitations in proficiency and cognitive demands.

***Prediction 1***

Given that the participants in the present study are advanced learners of Japanese with study-abroad experience, and that the materials are narrative texts likely to elicit perceptual simulation, we predict that all five situational dimensions—protagonist, time, space, causality, and intentionality—will significantly predict verb-clustering performance. Such a pattern would indicate a relatively high degree of multidimensional activation in situation model construction among L2 readers in this context.

***Simulation Hypothesis***

Grounded cognition theories propose that language comprehension involves simulating perceptual and motor experiences associated with described events (Zwaan 2004). From this view, individual differences in visual imagery should influence how effectively readers track and integrate situational dimensions. Prior research has shown that readers with more vivid imagery are more likely to construct detailed mental simulations, which in turn facilitates comprehension processes (e.g., Fincher-Kiefer 2001; Tang 2024).

***Prediction 2***

We predict that L2 readers with more vivid visual imagery will show stronger clustering effects for visually grounded dimensions, such as protagonist and space, compared to more abstract or linguistically mediated dimensions, such as time, causality, and intentionality.

In line with these objectives, this study investigates how Chinese learners of Japanese construct multidimensional situation models during L2 narrative reading, thereby contributing to a deeper understanding of the cognitive and individual factors that shape discourse-level processing.

## Methods

### Participants

This study recruited 29 advanced learners of Japanese whose L1 is Chinese, including 10 males and 19 females, with an average age of 26.14 years. The participants had an average Japanese learning duration of 6 years and 2 months, and an average study-abroad experience in Japan of 2 years and 5 months. All participants had passed the Japanese Language Proficiency Test (JLPT) N1, with an average reading section score of 46.31 out of 60 (*SD* = 10.10). This study was approved by the *** (Approval No. ***). Participants were compensated with a fee of 60 RMB upon completion of the experiment.

### Reading test materials and comprehension questions

In the process of L2 acquisition, learners frequently engage with narratives as a representative literary genre. Given the moderate difficulty level and the unlikelihood that participants would encounter entirely unfamiliar content at this stage, excerpts from Murakami’s (2021) *Birthday* *Girl *were selected as the primary experimental material (Table 1), comprising a total of 806 characters. The readability and difficulty of these excerpts were assessed using the jReadability Portal (https://jreadability.net/sys/en) and categorized as intermediate.

**Table 1:** Experimental material (*Birthday Girl*)

二十歳の誕生日、彼女はふだんと同じようにウェイトレスの仕事をした。金曜日はいつも彼女の受け持ちだったが、本来であればその金曜日の夜は仕事に出なくていいはずだった。もう一人のアルバイトの女の子に日にちを*交換してもらった*のだ。それはそうだ。コックに*怒鳴られ*ながら、かぼちゃのニョッキやら海の幸のフリットをテーブルまで*運ぶ*のは、二十歳の誕生日のまともな過ごし方とはいえない。でも、仕事を*代わってくれる*はずの女の子 が、風邪をこじらせて*寝こん*でしまった。四十度近く熱があり、下痢も*止まらない*ので、とても仕事ができる状態ではない、ということだった。それで急遽、彼女が*出勤する*ことになった。 「別に*気にしな*くていいのよ。」と彼女は電話口で、むしろ*詫びる*相手を*慰める*ように言った。「二十歳の誕生日だから特に何があるってわけでもないんだから。」 実際のところ、彼女はそれほどがっかりもしなかった。一緒に誕生日の夜を*過ごす*はずだったボーイフレンドと、数日前に深刻なけんかをしたこともその理由の一つだ。高校時代からずっと*交際していた*相手で、けんかの原因はたいしたことではなかった。しかし思いもかけず話がこじれ、売り言葉に買い言葉で激しい口論がひとしきり続いたあと、それまで二人をつないでいた絆が致命的に*損なわれてしまった*という感覚があった。彼女の中で石のように硬くなって*死んでしまった*ものがあった。けんかのあと彼からの連絡はなかったし、彼女のほうから*電話する*気にもならなかった。 彼女が働いていたのはそこそこに名の知れた六本木のイタリア料理店だった。六〇年代半ばからやっている店で、出てくる料理には先端的な鋭さはなかったが、味自体は至極まっとうなもので、*食べ飽きる*ことがなかった。店の雰囲気にも押しつけがましいところがなく、穏やかな落ち着きが*感じられていた*。若い客よりは年配の常連客が多く、場所がらその中には有名な俳優や作家も*交じっていた*。
English translation On her twentieth birthday, she worked as a waitress as usual. Friday was always her shift, but originally, she was not supposed to work that night. She had arranged to swap shifts with another part-time waitress. It made sense—getting yelled at by the cook while carrying plates of pumpkin gnocchi and seafood fritto to tables was hardly an ideal way to spend one’s twentieth birthday. However, the girl who was supposed to take her shift came down with a severe cold. With a fever approaching 40 degrees and persistent diarrhea, she was in no condition to work. As a result, the protagonist had to step in at the last minute. “Don’t worry about it,” she said over the phone, comforting the other girl, who was apologizing profusely. “It’s not like I had anything special planned for my twentieth birthday anyway.” In truth, she was not particularly disappointed. One reason was that she had had a serious argument with her boyfriend a few days prior, ruining their plans to spend the evening together. They had been dating since high school, and the cause of the fight was nothing serious. However, the conversation unexpectedly got out of hand, and after a heated argument that turned into a sales pitch, there was a sense that the bond that had held them together had been fatally damaged. There was something inside her that had hardened and died like a stone. She had not heard from him since the quarrel, nor did she feel inclined to call him. She was working at a well-known Italian restaurant in Roppongi. The restaurant had been in business since the mid-1960s, and although the food was not cutting-edge, the taste itself was quite decent, and one never got tired of eating it. The atmosphere of the restaurant was not intrusive, but rather calm and relaxed. There were more elderly patrons than young ones, and because of the location, some famous actors and writers were among them.

The target verbs in the excerpt above are: 交換してもらう (to have someone switch shifts), 怒鳴られる (to be yelled at), 運ぶ (to carry), 代わってくれる (to cover for someone), 寝こむ (to be sick in bed), 止まらない (to be unstoppable), 出勤する (to go to work), 気にしない (to not mind), 詫びる (to apologize), 慰める (to console), 過ごす (to spend time), 交際する (to date), 損なわれる (to be damaged), 死ぬ (to die), 電話する (to call), 食べ飽きる (to get tired of eating), 感じられる (to be felt), 交じる(to mix in)

As this study employed a verb-clustering paradigm to evaluate learners’ construction of multidimensional situation models, minor modifications were made to the original text. Specifically, two researchers collaboratively revised certain portions to ensure more balanced spacing between target verbs, thereby avoiding configurations in which all verbs were either overly clustered or excessively dispersed—patterns that could potentially interfere with clustering performance.

Additionally, six comprehension questions were designed to assess participants’ understanding of the text, with the correct answer to each question worth one point for a maximum total score of six. Following Wada (2019), participants with a comprehension accuracy below 50% (i.e., scores lower than 3) would not be included in the data analysis.

Finally, to help participants understand experimental procedures, excerpts from Ba’s (1948) novel *The Family* were selected as reading materials for the practice session, totaling 473 characters in length. The difficulty level of these materials was comparable to that of the primary experimental text.

### Situational analysis of verbs

To assess how readers mentally organize narrative events across multidimensional situation models, this study employed the verb-clustering task following the procedures of Zwaan et al. (1995b); Iseki and Kawasaki (2006). Prior to the experiment, 18 target verbs were selected, each appearing only once in the text. Following Iseki and Kawasaki (2006), all verbs were normalized to their base forms (e.g., past tense to infinitive), while voice (e.g., passive) and polarity (affirmative/negative) were left unchanged. To minimize the potential influence of orthographic variation on native Chinese participants (e.g., Cai et al. 2025; Fei et al. 2022; Song et al. 2023), all target verbs were consistently presented in *Kanji*.

All possible pairwise combinations of the 18 target verbs yielded 153 unique verb pairs. Each pair was independently evaluated for continuity along five situational dimensions: protagonist, space, time, causality, and intentionality. Two researchers rated the verb pairs independently, achieving an agreement rate of 81.9%. Disagreements were resolved through discussion, and consensus scores were used in the analysis. Specific examples of the coding criteria for each dimension are provided in Table 2.

**Table 2:** Evaluation criteria and examples for each variable

variable	Example
Protagonist	出勤する (to go to work) and 慰める (to console) are both actions of the protagonist (1 point), whereas 出勤する and 詫びる (to apologize) describe actions of the protagonist and a colleague, respectively (0 points).
Spatial	運ぶ (to carry) and 出勤する both take place in the restaurant (1 point), whereas 運ぶ and 寝こむ (to be sick in bed) occur in different locations (0 points).
Time	詫びる and 慰める happen in close succession (1 point), whereas 運ぶ and 慰める occur at different times (0 points)
Causality	詫びる and 慰める are causally related—without the colleague’s apology, the protagonist would not have consoled them (1 point). In contrast, 運ぶ and 慰める do not share a direct causal relationship (0 points).
Intentionality	交換してもらう (to have someone switch shifts) and 過ごす (to spend time) are linked because the protagonist switched shifts to celebrate their birthday (1 point), whereas 出勤する is not directly related to 過ごす as a intentionality (0 points).
Surface Connections	代わってくれる (to cover for someone) and 寝こむ are appear in the same sentence, while 代わってくれる and 出勤する are not in the same sentence.
Surface Distance	There is one meaningful words, 相手(the other side), between 詫びる and 慰める.

The evaluation criteria for each dimension were as follows:

- Protagonist: If both verbs described actions performed by the same protagonist, they were assigned a score of 1; otherwise, 0;
- Space: If the events occurred in the same location, a score of 1 was assigned; otherwise, 0;
- Time: If the events occurred within the same time frame, they received a score of 1; otherwise, 0;
- Causality: If one event was judged necessary for the occurrence of the other, the pair received a score of 1; otherwise, 0;
- Intentionality: If the two verbs were part of the same intentional action plan, a score of 1 was assigned; otherwise, 0.

In addition, following the framework of Zwaan et al. (1995b), verb clustering may be influenced not only by the situational coherence of events but also by their surface and semantic relationships. Consequently, this study included three fixed-effect control variables: surface connections, surface distance, and semantic similarity.

Surface connection was defined as whether both verbs appeared within the same sentence. Verb pairs that occurred in the same sentence were assigned a score of 1; otherwise, they received a score of 0. Surface distance was operationalized as the number of meaningful words (e.g., nouns, verbs, adjectives) occurring between the two verbs in the text. Semantic similarity was assessed by a separate cohort of 17 advanced Japanese learners (all of whom had passed the JLPT N1), who rated each verb pair on a five-point scale (1 = completely dissimilar, 5 = highly similar). The average rating for each pair was then computed as its semantic similarity score.

### Materials for the visual imagery vividness questionnaire

To assess visual imagery vividness, the Chinese version of the Vividness of Visual Imagery Questionnaire (VVIQ-C)—a culturally adapted and psychometrically validated instrument—was employed (Zhang et al. 2024, preprint). The scale ranges from 16 to 80 points and was developed following international translation guidelines (Muñiz et al. 2013), including independent forward and backward translations and expert panel review. Validation with a sample of 297 participants demonstrated excellent reliability, with strong internal consistency (Cronbach’s α = 0.92), split-half reliability (*r* = 0.82), and test-retest reliability over three months (*r* = 0.93). These values meet or exceed conventional psychometric standards. Moreover, its applicability was confirmed in a large-scale study involving 1,015 participants aged 8 to 60 years. Given its rigorous development and validation, the VVIQ-C is a reliable tool for measuring subjective mental imagery vividness in Chinese-speaking populations.

The questionnaire includes four everyday scenarios, i.e., “relatives or friends seen frequently,” “the scene of the sun rising,” “shops visited frequently,” and “a scene with trees, hills, and a lake,” comprising a total of 16 items. Participants were asked to visualize the corresponding scenes or images in their minds and rate the vividness of these mental images. The rating scale is as follows: 1 point for “no image at all,” 2 points for “a blurry image,” 3 points for “a barely clear and vivid image,” 4 points for “a moderately clear and vivid image,” and 5 points for “a very clear and vivid image.”

### Experimental procedures

**Fig. 1:**
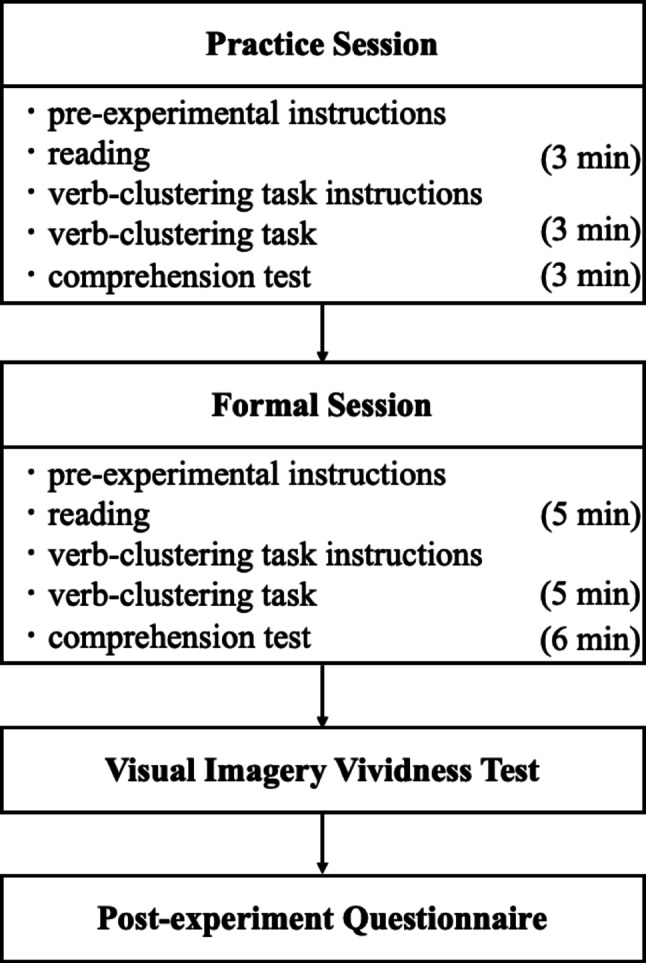
Experimental Procedures

The experiment comprised four parts: a practice session, formal reading, a post-experiment questionnaire, and the VVIQ-C test. The practice session and formal reading materials were provided in printed booklets, while the questionnaire and VVIQ-C test were administered online. The experimental procedure (Fig. 1) was as follows:

- Practice Session: Before reading, participants were instructed that they could re-read the material within the allotted time but were not allowed to take notes. After the 3-minute reading period, they completed a verb-clustering task (3 min) followed by a comprehension test (3 min). In the verb-clustering task, participants received test booklets with the target verbs and several response boxes. They were asked to group verbs based on their memory of the story without explicitly stating their grouping criteria. Before completing the task, they were given the following instructions: “During the verb-clustering task, you may not refer back to the text. Please group verbs you consider to belong together in the same response box while placing different groups in separate boxes. Use all the provided verbs, ensuring that each verb is used only once. You may use as many response boxes as needed and there is no limit to the number of verbs that can be placed in each box.”
- Formal Session: The procedure was identical to the practice session. Participants read the experimental text within the 5-minute time limit, followed by the 5-minute verb-clustering task and the 6-minute comprehension test.
- Visual Imagery Vividness Test: Participants completed the VVIQ-C.
- Post-experiment Questionnaire: Participants filled out a questionnaire that collected demographic information, Japanese learning history, and study-abroad experience.

## Results

### Data processing and analysis methods

To address RQ1, verb-clustering scores (0 or 1) were treated as the dependent variable. As described in the *Experimental Procedures* section, participants placed verbs they believed belonged to the same category within the same response box, based solely on their understanding of the material, without explicit instructions on categorization criteria. When scoring the verb-clustering task, the researchers referenced the 153 verb pairs (see the *Situational Analysis of Verbs* section). A score of 1 was assigned if a participant grouped a verb pair into the same cluster based on their subjective judgment; conversely, a score of 0 was assigned if the verbs were categorized into separate clusters. The clusters generated by participants were then compared against judgments made by two researchers based on the five situational dimensions, to assess the extent to which participants constructed dimensionally coherent situation models during reading.

The five dimensions (i.e., protagonist, space, time, causality, and intentionality), along with surface-level features (i.e., surface connections, surface distance, and semantic similarity), were included as fixed effects. Verb pairs (items) and participants were treated as random intercepts. A mixed-effects logistic regression model was implemented using the *GAMLj* module (Gallucci 2019) in *Jamovi* (version 2.6.2), which internally relies on the *glmer* function from the lme4 package in R (R Core Team 2024).

To address RQ2 and to facilitate model convergence and reporting clarity, only the significant predictors identified in the RQ1 analysis were retained as fixed effects. In addition, participants’ standardized VVIQ-C scores and their interactions with the selected dimensions were included as fixed effects. As in RQ1, verb pairs and participants were modeled as random intercepts. The same logistic mixed-effects regression model was employed using the *GAMLj* module in *Jamovi* to examine how the selected dimensions were modulated by individual differences in visual imagery vividness.

### Results for research question 1

The average score for task comprehension was 4.38 (*SD* = 0.77), with all participants scoring above 3, thus all were included in the analysis. The results of the mixed-effects logistic regression analysis are shown in Table 3. The results indicated that the dimensions of protagonist (*p* < 0.001), space (*p* < 0.001), time (*p* < 0.001), surface distance (*p* < 0.001), and semantic similarity (*p* = 0.024) significantly affected the verb-clustering scores, whereas the dimensions of intentionality (*p* = 0.746), causality (*p* = 0.304), and surface connections (*p* = 0.701) did not have a significant impact on verb-clustering performance.

**Table 3:** Analysis results for research question 1

Fixed Effects Parameter Estimates					
Names	Effect	Estimate	SE	z	p
(Intercept)		-1.05	0.38	-2.77	0.006
Protagonist	*Y - N*	0.91	0.16	5.81^****^	< 0.001
Spatial	*Y - N*	0.74	0.20	3.79^****^	< 0.001
Time	*Y - N*	0.60	0.16	3.73^****^	< 0.001
Causality	*Y - N*	-0.07	0.22	-0.33	0.746
Intentionality	*Y - N*	0.56	0.55	1.03	0.304
Surface Connections	*Y - N*	0.17	0.44	0.38	0.701
Surface Distance		-0.78	0.08	-9.54^****^	< 0.001
Semantic Similarity		0.15	0.07	2.26^*^	0.024

Random Components					
Groups	Name	SD	Variance	ICC	
Item	(Intercept)	0.60	0.36	0.10	
Participant	(Intercept)	0.76	0.57	0.15	

Item = 153, participant = 29, total observation = 4,437

The model is glmer (acc ~ protagonist + spatial + time + causality + intentionality + surface connections + surface distance + semantic similarity + (1 | item) + (1 | participant), family = binomial)

^†^*p* < 0.10, ^*^ *p* < 0.05, ^**^p < 0.01, ^***^p < 0.005, ^****^p < 0.001

### Analysis results for research question 2

The results of RQ1 indicate that the dimensions of protagonist, space, and time had significant effects. Therefore, this section further investigates how these three dimensions were influenced by participants’ visual imagery vividness.

#### Interaction effect of the protagonist dimension and visual imagery vividness

The results of the mixed-effects logistic regression analysis are shown in Table 4. The interaction between the protagonist dimension and visual imagery vividness was significant (*p* < 0.001). Simple effects tests revealed that when the target verbs referred to actions performed by different protagonists, visual imagery vividness significantly influenced verb-clustering scores (*p* = 0.030). Specifically, higher visual imagery vividness was associated with greater activation of the protagonist dimension. However, when the target verbs referred to actions performed by the same protagonist, visual imagery vividness had no significant effect on verb-clustering scores (*p* = 0.970).

**Table 4:** Interaction effect of protagonist dimension and visual imagery vividness

Fixed Effects Parameter Estimates					
Names	Effect	Estimate	SE	z	p
(Intercept)		-1.61	0.18	-8.88	< 0.001
Protagonist	Y-N	1.49	0.24	6.29^****^	< 0.001
VVIQ-C	VVIQ-C	0.16	0.15	1.10	0.273
Protagonist × VVIQ-C	Y-N × VVIQ-C	-0.33	0.10	-3.44^****^	< 0.001

Random Components					
Groups	Name	SD	Variance	ICC	
Item	(Intercept)	1.13	1.27	0.28	
Participant	(Intercept)	0.74	0.54	0.14	

Item = 153, participant = 29, total observation = 4,437

The model is glmer (acc ~ protagonist + VVIQ-C + protagonist: VVIQ-C + (1 | item) + (1 | participant), family = binomial)

^†^*p* < 0.10, ^*^ *p* < 0.05, ^**^p < 0.01, ^***^p < 0.005, ^****^p < 0.001

#### Interaction effect of the space dimension and visual imagery vividness

The results of the mixed-effects logistic regression analysis are shown in Table 5. The interaction between the space dimension and visual imagery vividness was significant (*p* = 0.007). A simple effects test revealed that when the space dimension was discontinuous, the effect of visual imagery vividness on verb-clustering performance was marginally significant (*p* = 0.066). This suggests that participants with higher visual imagery vividness tended to activate the space dimension even in the absence of spatial continuity. However, when the space dimension was continuous, visual imagery vividness did not significantly affect verb-clustering performance (*p* = 0.914).

**Table 5:** Interaction effect of Spatial dimension and visual imagery vividness

Fixed Effects Parameter Estimates					
Names	Effect	Estimate	SE	z	*p*
(Intercept)		-1.66	0.20	-8.49	< 0.001
Spatial	*Y-N*	1.04	0.28	3.73^****^	< 0.001
VVIQ-C	*VVIQ-C*	0.13	0.15	0.86	0.388
Spatial × VVIQ-C	*Y-N × VVIQ-C*	-0.29	0.11	-2.70^**^	0.007

Random Components					
Groups	Name	SD	Variance	ICC	
Item	(Intercept)	1.22	1.48	0.31	
Participant	(Intercept)	0.73	0.54	0.14	

Item = 153, participant = 29, total observation = 4,437

The model is glmer (acc ~ spatial + VVIQ-C + spatial: VVIQ-C + (1 | item) + (1 | participant), family = binomial)

^†^*p* < 0.10, ^*^ *p* < 0.05, ^**^p < 0.01, ^***^p < 0.005, ^****^p < 0.001

#### Interaction effect of the time dimension and visual imagery vividness

The results of the mixed-effects logistic regression analysis are shown in Table 6. The interaction between the time dimension and visual imagery vividness was not significant (*p* = 0.115). This indicates that visual imagery vividness did not significantly affect verb-clustering performance across different levels of time continuity.

**Table 6:** Interaction effect of time dimension and visual imagery vividness

Fixed Effects Parameter Estimates					
Names	Effect	Estimate	SE	z	p
(Intercept)	(Intercept)	-1.76	0.18	-9.91	< 0.001
Time	Y-N	1.14	0.23	5.07^****^	< 0.001
VVIQ-C	VVIQ-C	0.19	0.14	1.28	0.199
Time × VVIQ-C	Y-N × VVIQ-C	-0.15	0.09	-1.58	0.115

Random Components					
Groups	Name	SD	Variance	ICC	
Item	(Intercept)	1.15	1.33	0.29	
Participant	(Intercept)	0.73	0.53	0.14	

Item = 153, participant = 29, total observation = 4,437

The model is glmer (acc ~ time + VVIQ-C + time: VVIQ-C + (1 | item) + (1 | participant), family = binomial)

^†^*p* < 0.10, ^*^ *p* < 0.05, ^**^p < 0.01, ^***^p < 0.005, ^****^p < 0.001

#### Post hoc power analysis for mixed-effects models

To further evaluate whether the study was adequately powered to detect theoretically relevant effects, we conducted post hoc power analyses for the three generalized linear mixed-effects models testing the interaction between VVIQ-C and task condition (protagonist, space, and time). For each model, the fixed-effect coefficient corresponding to the interaction term was extracted and converted into an approximate standardized effect size (*Cohen’s d*), following the transformation recommended for logistic regression coefficients (cf. Chinn 2000).

Power estimation was conducted using the Crossed Power App (https://jakewestfall.shinyapps.io/crossedpower/), which implements the simulation-based procedure developed by Westfall et al. (2014) for crossed random-effects designs. This method explicitly accounts for the nested structure of the data by partitioning variance across residual error, participant-level random intercepts, stimulus-level random intercepts, and participant-by-stimulus interactions. By incorporating these variance partitioning coefficients (VPCs), the approach yields robust power estimates in complex experimental designs.

The estimated effect sizes for the interaction terms were *d* = 0.18 for protagonist × VVIQ-C, *d* = 0.16 for space × VVIQ-C, and *d* = 0.10 for time × VVIQ-C. Despite the relatively small sample of participants (*N* = 29), the fully crossed structure with a large number of stimuli (*n* = 153) yielded high statistical power across all models (power ≈ 1.00). These results suggest that the present study was sufficiently powered to detect modest interaction effects involving individual difference measures.

## Discussion

This study drew on the verb-clustering task developed by Zwaan et al. (1995b) to examine how Chinese learners of Japanese construct situation models while reading in their L2. A key contribution is that we considered the moderating role of visual imagery vividness, an issue that has received little attention in previous research. Specifically, the study addressed two questions: RQ1 focused on how learners represent five situational dimensions (protagonist, time, space, causality, and intentionality) during narrative comprehension; RQ2 examined whether individual differences in visual imagery vividness shape this process. In what follows, we respond to each question in turn and discuss the broader implications for theories of reading comprehension and L2 pedagogy, with particular attention to Asian language pairings.

### Construction of situation models during the L2 Japanese reading process by Chinese learners

RQ1 examined how Chinese learners of Japanese construct situation models across five dimensions—protagonist, time, space, causality, and intentionality—while reading L2 Japanese narratives. A mixed-effects logistic regression analysis was conducted to examine the influence of these dimensions on verb-clustering scores. The results revealed that the protagonist, space, and time dimensions, as well as surface distance and semantic similarity, significantly predicted clustering outcomes. In contrast, the causality and intentionality dimensions, along with surface connections, did not exert significant effects. These findings provide partial support for the *Event Indexing Hypothesis*, indicating that while L2 readers activated multiple situational dimensions during narrative comprehension, the degree of activation varied across dimensions.

These findings show both similarities and differences compared to previous research. Zwaan and Brown (1996) found that L2 readers mainly monitored the protagonist, time, and causality dimensions. In contrast, Takaki (2010) reported sensitivity to the protagonist and space dimensions. In our study, Chinese learners of Japanese paid close attention to protagonists and successfully integrated temporal and spatial information while reading Japanese narratives. This suggests that the protagonist, space, and time dimensions are particularly important for this group. Among them, the protagonist dimension appears to be consistently important across different studies. This may be attributed to the fact that the protagonist dimension typically constitutes the central focus of the narrative and conveys the most salient information around which the story is structured (Ushiro et al. 2022). However, findings on time and space remain inconsistent. One possible reason lies in the differences in the type of reading materials. This study used narratives, where temporal and spatial elements are often more prominent and easier to track. In addition, certain similarities between Chinese and Japanese, such as the positioning of temporal adverbs (e.g., Tamaoka and Zhang 2022) and the use of spatial demonstratives (Zhao et al. 2024), may facilitate learners’ monitoring of time and space dimensions during L2 reading.

The absence of significant effects for the causality and intentionality dimensions on verb-clustering scores aligns with the findings of Takaki (2010). As Zwaan and Radvansky (1998) have argued, those two dimensions are not primarily involved in identifying individual events but rather in discerning relationships between them. In particular, the intentionality dimension—which encompasses intentional actions and planning—often requires readers to engage in higher-order reasoning and to integrate multiple sources of information. Compared to native readers, L2 readers typically generate fewer explanatory inferences (Zwaan and Brown 1996), which may hinder their ability to accurately perceive causal and intentional links between events. This cognitive challenge may explain why L2 readers find it more difficult to effectively monitor the causality and intentionality dimensions during narrative comprehension.

Surface distance and semantic similarity were also found to significantly influence verb-clustering scores. The negative effect of surface distance suggests that as the linguistic distance between verbs increases, the accuracy of situation model construction declines. This may reflect the increased cognitive load associated with integrating information across more distant textual elements, which heightens the risk of processing errors. In contrast, the positive effect of semantic similarity indicates that verbs with higher semantic similarity are more likely to be categorized together. This finding aligns with previous studies (Zwaan et al. 1995b; Takaki 2010; Wada 2019), which similarly reported that semantically related verbs are more easily associated. Taken together, these results suggest that surface distance and semantic similarity are key factors affecting the accuracy of verb clustering in the context of situation model construction.

### The impact of visual imagery vividness on the construction of situation models in L2 reading

RQ2 investigated how the vividness of visual imagery influences Chinese learners’ construction of situation models across multiple dimensions. The results revealed significant interaction effects between visual imagery vividness and both the protagonist and space dimensions, while no significant interaction effect was observed for the time dimension. These findings are largely consistent with the *Simulation Hypothesis*, suggesting that readers with more vivid visual imagery are better able to simulate visually grounded dimensions such as protagonist and space, whereas temporal information is less dependent on imagery processes.

First, the interaction between visual imagery vividness and the protagonist dimension was significant. When the target verbs described actions performed by different protagonists, visual imagery vividness had a pronounced effect on verb-clustering scores. Higher visual imagery vividness was associated with stronger activation of the protagonist dimension. In contrast, when the verbs referred to actions performed by the same protagonist, visual imagery vividness did not exert a significant influence. This finding aligns with Denis (1982), who proposed that readers with higher visual imagery vividness tend to form more complex and detailed mental images. Therefore, when confronted with actions by different protagonists, learners with vivid visual imagery were better able to use mental images to fill informational gaps, resulting in more accurate construction of situation models.

Moreover, the interaction between visual imagery vividness and the space dimension was also significant. Readers with higher visual imagery vividness tended to activate the space dimension, even when the target verbs did not occur within the same spatial context. This finding highlights the role of visual imagery vividness in processing discontinuous spatial information. Learners with vivid visual images appeared better equipped to mentally bridge spatial discontinuities, thereby enhancing the coherence of their situation models.

In contrast, the interaction between visual imagery vividness and the time dimension was not significant, suggesting that visual imagery vividness did not meaningfully influence verb-clustering scores across varying levels of temporal continuity. Temporal information is inherently abstract and less imageable, making it less amenable to perceptual simulation than more concrete dimensions such as space or protagonists. This abstract quality leads to weaker mental imagery activation during temporal processing, which may explain why individual differences in visual imagery vividness show limited effects for the time dimension. Supporting this view, Boroditsky and Ramscar (2002) demonstrated that temporal representations rely on metaphorical mappings from spatial concepts rather than direct perceptual grounding, reinforcing that temporal abstraction diminishes dependence on sensory-based mental simulation.

In conclusion, visual imagery vividness plays a critical role in the construction of situation models. It facilitates coherence maintenance when readers encounter shifts in protagonists or spatial dimensions particularly relevant to L2 narrative comprehension. This finding aligns with previous studies (e.g., Denis 1982, 1987; Tang 2024), which found that individuals with more vivid mental imagery demonstrate superior text understanding and recall. Similarly, Atoum and Reziq (2018) and Devi and Sia (2020) highlighted visual imagery as a significant predictor of reading comprehension. Building on foundational research (e.g., Zwaan and Brown 1996; Takaki 2010), the present study advances the literature by showing that imagery vividness selectively modulates specific dimensions of situation model construction in L2 reading—an effect not previously documented. These results suggest that integrating imagery-based strategies into L2 pedagogy may enhance learners’ ability to build coherent mental representations, thereby improving overall comprehension.

## Conclusions, limitations and future directions

### Conclusions

This study addressed two primary research questions: (1) How do Chinese learners of Japanese construct multidimensional situation models during L2 narrative reading? and (2) To what extent does visual imagery vividness influence this process? The results showed that three dimensions—protagonist, time, and space—played particularly salient roles in situation model construction. Moreover, learners with higher visual imagery vividness formed more coherent mental representations, especially when processing shifts in the protagonist and space dimensions.

These findings demonstrate that visual imagery vividness selectively supports situation model construction in L2 reading, particularly for visually grounded dimensions. By revealing dimension-specific effects, the study contributes to a deeper understanding of how individual cognitive differences shape L2 text comprehension. Pedagogically, the results suggest that incorporating imagery-based strategies into instruction may help learners build richer and more coherent mental representations, thereby enhancing reading comprehension.

### Limitations and future directions

Despite these contributions, several limitations should be acknowledged.

First, regarding the experimental design, this study focused exclusively on L2 narrative reading. While prior studies have employed the verb-clustering paradigm in L2 contexts (e.g., Takaki 2010), only Zwaan and Brown (1996) directly compared situation model construction in L1 and L2 within the same participants. Designing such comparisons requires carefully matched texts across discourse structure, thematic content, difficulty, and cultural familiarity—an objective future research should pursue to enable more robust cross-linguistic comparisons.

Second, regarding the participant sample, the relatively small sample size (*N* = 29), although supported by post hoc power analysis, limits the generalizability of the findings. Moreover, all participants were advanced learners of Japanese with study-abroad experience. While this helped control for language proficiency, it restricts applicability to learners at other developmental stages. Future studies should increase sample size, include learners across different proficiency levels, and consider longitudinal or within-participant designs to track how situation model construction evolves over time and across L1–L2 contexts.

Third, regarding variable selection, the study primarily examined visual imagery vividness as an individual-difference factor. Although the results demonstrated its selective influence on visually grounded dimensions, other cognitive factors—such as executive function, working memory, or attentional control—may also shape L2 situation model construction. Future research should incorporate these additional variables to build a more comprehensive account. Additionally, the absence of significant effects for the causality dimension raises questions. Given prior evidence that visual aids (e.g., illustrations) can facilitate causal inference (Wada 2019), future studies might explore whether such scaffolding can support causal integration in L2 readers.

## Data Availability

The datasets used and/or analyzed during the current study are available from the corresponding author on reasonable request.
